# Single-leg balance in “instability” footwear

**DOI:** 10.1186/1757-1146-5-S1-P10

**Published:** 2012-04-10

**Authors:** Carina Price, Laura Smith, Philip Graham-Smith, Richard Jones

**Affiliations:** 1 Centre for Health Sciences Research, University of Salford, Salford, Greater Manchester, M6 6PU, UK

## Background

The concept of instability footwear is to reduce stability, increase muscle activation and “tone”. Recently numerous brands have developed instability footwear for significant sales. Despite extensive marketing claims there are few empirical studies quantifying effects of instability footwear on muscle activity or motion in healthy individuals aside from Masai Barefoot Technology (MBT^TM^) [1,2]. The aim of the study was to quantify instability in single-leg standing in a variety of commercially available instability sandals.

## Methods

Fifteen female subjects participated (age: 29±6.7 years, mass: 62.6±6.9 kg, height: 167.1±4.2 cm). The protocol quantified Centre of Pressure (CoP) excursion (Kistler) and lower extremity integrated muscle activity (IEMG) (Noraxon) for three thirty second single-leg standing trials in four experimental conditions and one control (Earth Footwear^TM^). The instability footwear conditions were FitFlop^TM^, MBT^TM^, Reebok Easy-Tone^TM^ and Skechers Tone-Ups^TM^. IEMG is presented normalised to control.

## Results

Repeated measures ANOVA revealed significant differences in CoP with MBT having significantly greater anterior-posterior range than Control (*p*=0.012), FitFlop (*p*=0.033) and Skechers (*p*=0.014) (Table 1). Medial-lateral ranges were consistent between conditions. Testing identified increased CoP velocity in anterior-posterior and medial-lateral directions in MBT compared to other conditions, but neither reached significance. IEMG was higher in instability shoes with average increases for gastrocnemius (44%) and peroneals (18%). The only statistical IEMG difference was gastrocnemius in Skechers with a 45% increase compared to control (*p*=0.042).

**Table 1:** CoP and IEMG results for the footwear conditions.

	Control	Fitflop	MBT	Reebok	Skechers
CoP medial-lateral range (mm)	36.5 (±7.8)	35.5 (±4.1)	34.9 (±3.8)	34.6 (±4.7)	34.0 (±4.3)
CoP anterior-posterior range (mm)	49.6 (±11.1)	53.0 (±8.4)^#^	64.0 (±10.9)*^,#^	50.3 (±15.0)	49.3 (±12.3)^#^
CoP medial-lateral velocity (mm.s^-1^)	29.8 (±4.8)	28.7 (±4.9)	30.0 (±6.1)	29.3 (±5.6)	28.5 (±6.2)
CoP anterior-posterior velocity (mm.s^-1^)	26.4 (±3.6)	27.7 (±4.7)	28.4 (±5.0)	27.9 (±4.9)	26.8 (±5.1)
Medial gastrocnemius IEMG (%)	-	1.37 (±0.52)	1.53 (±0.75)	1.39 (±0.64)	1.45 (±0.51)*
Peroneals IEMG (%)	-	1.19 (±0.33)	1.21 (±0.31)	1.15 (±0.22)	1.16 (±0.21)

* Denotes significant difference between control and instability condition (*p*<0.05)

^#^ Denotes significant difference between instability conditions (*p*<0.05)

## Conclusions

Increased anterior-posterior CoP range in MBT is expected due to the rocker profile [2]. Other conditions have footbeds with intrinsic instability not an external feature, which may increase effectiveness in gait. IEMG increased in experimental conditions showing instability shoes increased total activation, however high variability masks statistical differences. Inter-subject differences forms part of on-going analysis. Limitations of single-leg balance mimicking gait are recognised; increased duration of muscle activation is claimed by brands and fixed-duration testing negates this.
